# The value of combined hemodynamic, respiratory and intra-abdominal pressure monitoring in predicting acute kidney injury after major intraabdominal surgeries

**DOI:** 10.1080/0886022X.2019.1587467

**Published:** 2019-03-25

**Authors:** Csaba Kopitkó, László Medve, Tibor Gondos

**Affiliations:** a Intensive Care Unit, Dr. Kenessey Albert Hospital, Balassagyarmat, Hungary;; b Faculty of Health Sciences, Department of Clinical Studies, Semmelweis University, Budapest, Hungary

**Keywords:** Postoperative acute kidney injury, central venous pressure, intraabdominal pressure, mean airway pressure

## Abstract

**Background**: The incidence of postoperative acute kidney injury (AKI) is predominantly determined by renal hemodynamics. Beside arterial blood pressure, the role of factors causing a deterioration of venous congestion (intraabdominal pressure, central venous pressure, mechanical ventilation) has emerged. The value of combined hemodynamic, respiratory and intra-abdominal pressure (IAP) monitoring in predicting postoperative acute kidney injury has received only limited exploration to date.

**Methods**: Data were collected for adult patients admitted after major abdominal surgery at nine Hungarian ICUs. Hemodynamic parameters were compared in AKI vs. no-AKI patients at the time of admission and 48 h thereafter. Regarding ventilatory support, we tested mean airway pressures (Pmean). Effective renal perfusion pressure (RPP) was calculated as MAP−(IAP + CVP + Pmean). The Mann–Whitney *U* and the chi-square tests were carried out for statistical analysis with forward stepwise logistic regression for AKI as a dependent outcome.

**Results**: A total of 84 patients (34 ventilated) were enrolled in our multicenter observational study. The median values of MAP were above 70 mmHg, IAP not higher than 12 mmHg and CVP not higher than 8 mmHg at all time-points. When we combined those parameters, even those belonging to the ‘normal’ range with Pmean, we found significant differences between no-AKI and AKI groups only at 12 h after ICU admission (median and IQR: 57 (42–64) vs. 40 (36–52); *p* < .05). Below it’s median (40.7 mmHg) on admission, AKI developed in all patients. If above 40.7 mmHg on admission, they were protected against AKI, but only if it did not decrease within the first 12 h.

**Conclusions**: Calculated effective RPP with the novel formula MAP−(IAP + CVP + Pmean) may predict the onset of AKI in the surgical ICU with a great sensitivity and specificity. Maintaining effective RPP appears important not only at ICU admission but during the next 12 h, as well. Additional, larger studies are needed to explore therapeutic interventions targeting this parameter.

## Introduction

Acute kidney injury (AKI) associates with poor prognosis in critically ill patients [1–3]. There are well-known risk factors for postoperative AKI: (1) sepsis especially due to intraabdominal infections; (2) deterioration of renal blood supply; (3) type of surgery (intraabdominal, cardiac, emergency interventions); (4) mechanical ventilation; (5) vasopressor therapy; (6) preexisting kidney disease [1,4–9]. The deterioration of arterial blood supply as indicated by a mean arterial blood pressure (MAP) less than 65 mmHg clearly leads to worsening of kidney function, but until now only limited animal and human studies have been conducted for highlighting the role of elevated renal venous pressures [10–12].

Abdominal perfusion pressure (APP), recommended by the WSACS – the Abdominal Compartment Society for estimating the adequacy of renal perfusion, is computed by MAP – intraabdominal pressure (IAP) [13]. Several studies have been published on this method [14–18]. Elevated IAP with or without a decreased APP has been shown to be present in connection with a higher incidence of AKI [13]. On the other hand, other possible contributors to impair venous return and net renal perfusion pressures, such as central venous pressure (CVP) or transmitted airway pressures induced by mechanical ventilation have not been sufficiently considered to date. Several other intensive care investigators use the term ‘transrenal pressure’, defined as MAP – CVP as a predictor of AKI, albeit without considering the impact of IAP as a contributing factor [10–12]. To date, only one study has been conducted to investigate the relationship between elevated CVP and the rise of IAP; however, this study found poor correlation between elevated CVP and IAP in decompensated heart failure patients [19]. The WSACS – the Abdominal Compartment Society proposed the filtration gradient (FG = MAP − 2 × IAP) for computing the renal perfusion pressure in the Society’s former guidelines, but this recommendation was subsequently withdrawn in 2013 [13,20].

In daily practice, mechanical ventilation has a well-known association with a higher risk of AKI, but none of the individual respiratory parameters, such as positive end-expiratory pressure (PEEP), tidal volume, mean airway pressure (Pmean), either alone or combined with any circulatory pressure parameters has proven predictive with regard to AKI until now [21].

Proper hemodynamic monitoring improves outcomes after major abdominal surgery [22]. For this reason, the continuous measurement of MAP and IAP is generally accepted and widely practiced [13]. The majority of postoperative AKI cases are attributable to the impairment of the effective vascular perfusion to the kidney; therefore, the optimization of macro-hemodynamics remains critically important [9].

Most recently, we reported [MAP−(IAP +  CVP + Pmean)], a novel formula for predicting renal perfusion pressure (RPP) and calculated on admission to the ICU after major abdominal surgery, appears to be the most precise way to predict AKI in critically ill postoperative patients from the twelve investigated formulas [23]. The aim of our study was to test the hypothesis whether the formula, we termed ‘effective RPP’ that includes MAP, IAP, CVP and Pmean are appropriate for predicting the onset of AKI in surgical ICU patients after major abdominal surgery.

## Materials and methods

### Patients

Data were collected prospectively in adult patients admitted after major abdominal surgery in nine Hungarian Intensive Care Units (ICU) (4 universities, 4 regional and 1 city hospitals) in our multicenter observational between 5 January 2015 and 6 October in 2015 [23]. Ethical approval for this study was provided by the Hungarian Health Registration and Training Center, the national ethical committee (Ethical Committee N° 039320/2014/OTIG). Written informed consent was obtained from all study participants or granted by their legal guardians or next-of-kin. The ethical standards of the experiments were in accordance with the guidelines provided by the World Medical Association Declaration of Helsinki on Ethical Principles for Medical Research Involving Humans for Studies.

Patients were enrolled after major abdominal surgery requiring a minimum of 48-h ICU postoperative therapy. Patients under 18 years of age, those with an end-stage kidney disease, those having surgery with suprarenal cross-clamping or undergoing kidney or urinary bladder operations were excluded from the study. Patients enrolled in other clinical studies were also excluded. To collect data, we used an Excel-based data collection file and, included demographic and perioperative information, severity of illness on admission (Simplified Acute Physiology Score version II (SAPS II), Sepsis-related Organ Failure Assessment Score (SOFA)) and the outcome data (presence of AKI at 48 h) [24,25]. The internationally accepted Acute Kidney Injury Network (AKIN) criteria (both creatinine and urine output) were used for defining AKI [26]. We considered a preoperative serum creatinine level at baseline [23].

### Measurements

MAP, CVP and IAP were recorded by commercially available patient monitors at the time of admission and at 6, 12, 24 and 48 h thereafter. The measurement of IAP was performed by nursing staff via indwelling urinary catheters and according to the WSACS – the Abdominal Compartment Society guidelines. RPP was calculated at the given time points according to the novel formula of MAP−(IAP + CVP + Pmean) and the onset of AKI was detected at 48 h after ICU admission. The measurement of CVP was performed at the end of expiration. All pressures were given in mmHg (1 mmHg = 1.36 cmH_2_O).

### Statistical analysis

All values were presented as median with interquartile range (IQR). The median values of the different groups were compared using the Mann–Whitney *U*-test and the occurrence rates using the chi-square test. To test the sensitivity and specificity of changes of RPP between 6 and 12 h, a Receiver Operating Curve (ROC) analysis was performed for parameters discriminating AKI and no-AKI groups [Group 1: patients without AKI; Group 0: patients with AKI (grade 1, 2 or 3)]. Confidence intervals (95%) were calculated according to the formula described by Hosmer and Lemeshow. While CVP and airway pressures can influence IAP, their independency has been analyzed by linear regression analysis, as shown in our previous work [23]. Data were dichotomized regarding effective RPP, which demonstrated the best predictivity with reference to its previously published median of 40.7 mmHg (measured on admission) when comparing no-AKI and AKI groups at later time points.

To explore the relationship between abdominal perfusion pressure and the incidence of AKI, forward stepwise logistic regression was performed. All the basic hemodynamic parameters, Pmean and the effective RPP on admission, 6 h and 12 h, as well as all the changes between these time points were included. All the pressures (MAP, CVP, IAP, Pmean and RPP) were considered as independent predictors measured at the same time or interval, and the presence of AKI as the outcome parameter.

Our study had an 80% statistical power of effective RPP 12 h after admission between the AKI and no-AKI groups at a significance level of 0.05. For estimating the effect size of our study, we calculated Hedges’ g modified for small samples. For MAP−(IAP + CVP + Pmean) Hedges’ g values were 0.74 on admission, 1.02 at 12 h after admission, and 0.93 at the change between 6 and 12 h. Hedges’ g is considered to be high above 0.8. All differences were deemed to be significant if *p* ≤ .05. All analyses were performed by the SPSS statistical software package 23.0 (IBM SPSS, Armonk, NY, US).

## Results

A total of 84 patients were admitted to the ICU during the study period (type of surgery and number of cases – gastric: 9; bowel: 28; rectal: 19; hepatic: 3; pancreatic: 4; aortic: 13; trauma: 5 and miscellaneous others: 3). Between the AKI and no-AKI groups, there were no significant differences in age, gender and serum albumin levels. A higher incidence of sepsis, as well as higher serum lactate levels and severity-of-disease scores were found in the AKI group (Table 1).

**Table 1.** Demographics of patients at admission (median and IQR; case numbers and percentage).

	no-AKI (*n* = 45)	AKI (*n* = 39)	*p*
Male/female	30/15	19/20	NS
Age (years)	65.0 (60.0–76.5)	68.0 (59.0–79.0)	NS
SAPS II	33.0 (22.0–40.0)	49.5 (32.8–65.3)	<.01
SOFA	5.0 (2.0–10.0)	10.0 (5.8–11.8)	<.001
Non-renal SOFA	3.0 (1.0–7.0)	7.0 (4.0–9.0)	<.01
Albumin (g/L)	29.4 (24.0–35.5)	26.9 (22.1–32.1)	NS
Lactate (mmol/L)	1.5 (1.1–2.4)	2.4 (1.8–3.5)	<.01
BUN (mmol/L)			
Preop	7.0 (4.5–9.0)	8.7 (5.1–14.8)	NS
Admission	5.1 (3.9–7.3)	10.0 (5.5–15.9)	<.01
Creatinine (micromol/L)			
Preop	85 (70–104)	96 (73–137)	NS
Admission	73 (56–90)	127 (72–213)	<.001
Sepsis	3 (6.7%)	16 (41.0%)	<.001
Need of ventilation	15 (33.3%)	25 (64.1%)	<.05
ICU mortality	2 (4.4%)	9 (24%)	<.01
Hospital mortality	5 (11.1%)	13 (33.3%)	<.05
ICU length of stay (days)	2.0 (2.0–4.0)	5.0 (2.0–11.5)	<.001
Hospital length of stay (days)	11.0 (9.0–14.0)	13.0 (8.0–18.5)	NS

AKI: Acute Kidney Injury; BUN: Blood Urea Nitrogen; SAPS II: Simplified Acute Physiology Score version II; SOFA: Sepsis-related Organ Failure Assessment Score.

The MAP and the calculated effective RPP values were significantly lower in the AKI group (Table 2) on admission. No differences were found in airway pressures between the two groups. The change of RPP between time points is presented in Table 3.

**Table 2.** The values of parameters (mmHg, median (IQR)).

Parameters		*n*	No AKI	*n*	AKI	*p*
MAP	Admission	45	89 (77–100)	39	82 (65–91)	NS
	6 h	45	75 (66–85)	39	72 (67–85)	NS
	12 h	45	76 (68–87)	39	72 (69–82)	NS
	24 h	42	81 (71–91)	37	80 (70–88)	NS
	48 h	32	81 (75–101)	37	82 (70–90)	NS
IAP	Admission	45	10 (6–13)	39	11 (7–16)	NS
	6 h	45	10 (6–12)	39	9 (7–14)	NS
	12 h	45	11 (7–13)	39	11 (7–15)	NS
	24 h	39	9 (7–13)	36	12 (8–16)	< .05
	48 h	29	10 (7–14)	37	12 (8–17)	NS
CVP	Admission	45	6 (2–10)	39	6 (4–8)	NS
	6 h	45	5 (1–8)	39	7 (4–8)	NS
	12 h	45	5 (2–7)	39	6 (4–9)	NS
	24 h	42	6 (4–7)	37	8 (3–11)	NS
	48 h	31	7 (4–9)	37	7 (5–11)	NS
Pmean	Admission	12	9 (7–10)	22	9 (8–11)	NS
	6 h	7	8 (7–10)	19	10 (6–11)	NS
	12 h	6	7 (7–10)	17	9 (7–12)	NS
	24 h	4	7 (6–9)	18	10 (7–12)	NS
	48 h	3	6 (6–7)	16	8 (7–14)	NS
MAP-IAP	Admission	45	88 (69–93)	39	65 (49–85)	< .005
	6 h	45	66 (56–74)	39	62 (55–77)	NS
	12 h	45	66 (57–77)	39	61 (53–76)	NS
	24 h	41	72 (60–84)	37	64 (57–77)	NS
	48 h	32	72 (63–91)	37	69 (57–82)	NS
MAP-(IAP + CVP + Pmean)	Admission	12	55 (50–59)	22	41 (30–52)	< .005
	6 h	7	49 (39–55)	19	44 (40–60)	NS
	12 h	6	57 (42–64)	17	40 (36–52)	< .05
	24 h	4	53 (45–58)	18	31 (18–46)	NS
	48 h	3	55 (52–59)	16	36 (3–50)	NS

AKI: Acute Kidney Injury; CVP: Central Venous Pressure; IAP: Intraabdominal Pressure; MAP: Mean Arterial Pressure.

**Table 3.** The change of RPP in the first 12 h after admission to ICU (mmHg, median (IQR)).

Parameter		*n*	No AKI	*n*	AKI	*p*
Δ[MAP-(IAP + CVP + Pmean)]	0–6 h	8	−5 (−41 to –6)	23	6 (−9 to –18)	NS
	6–12 h	7	9 (2–13)	21	−4 (−10 to –1)	<.05
	0–12 h	7	3 (−28 to –14)	21	−6 (−14 to –11)	NS

**Figure 1.**
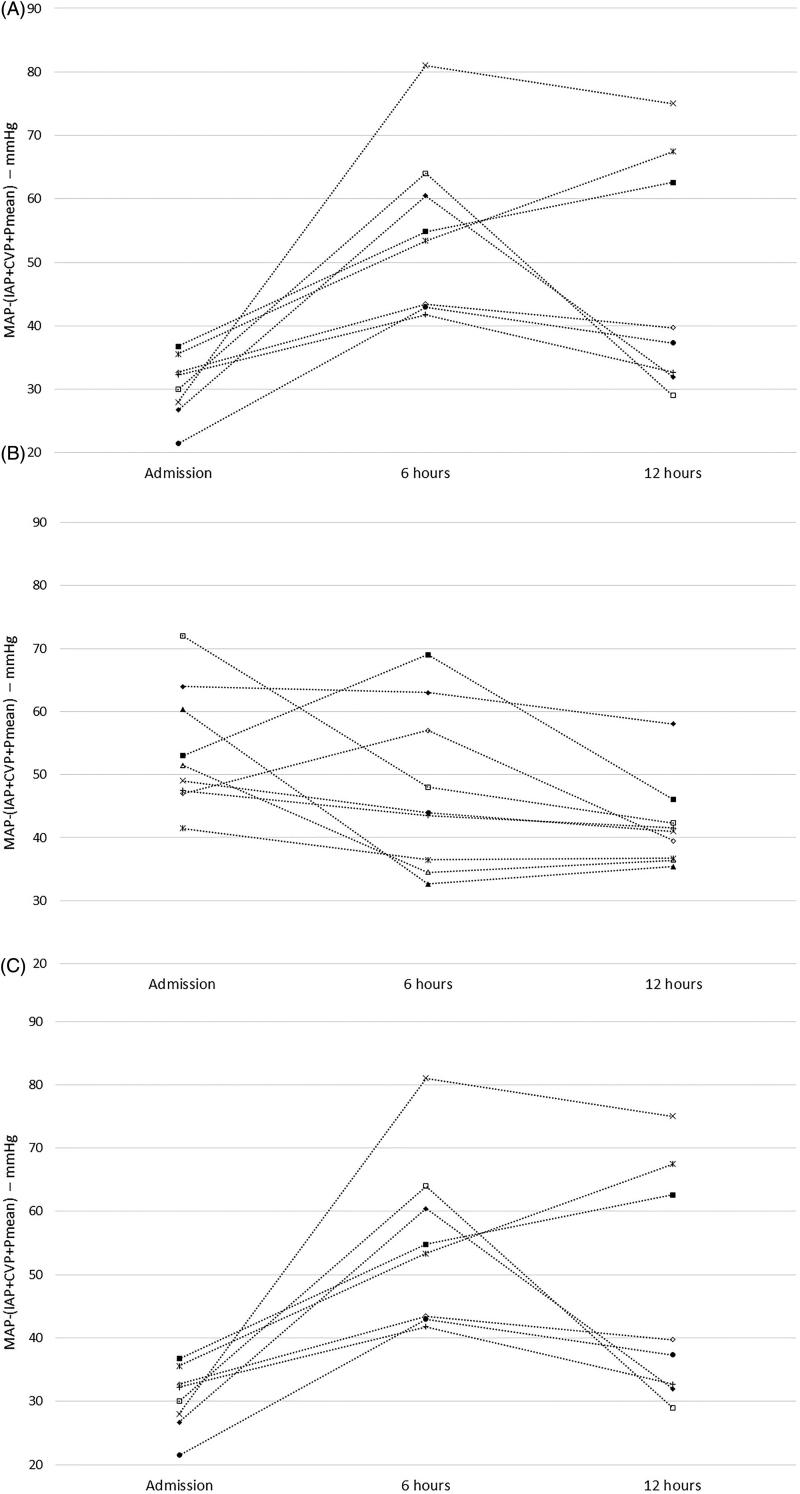
The patients' data with (A) below 40.7 mmHg of MAP−(IAP + CVP + Pmean) and above it (B) with or (C) without AKI in the first 12 h.

**Figure 2.**
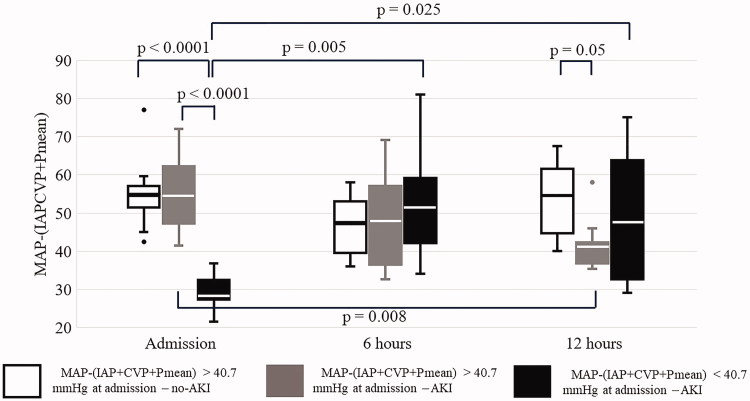
The course of MAP−(IAP + CVP + Pmean) in the first 12 h in ICU after major abdominal surgery. The *p* values within or between groups not shown were not statistically significant.

**Figure 3.**
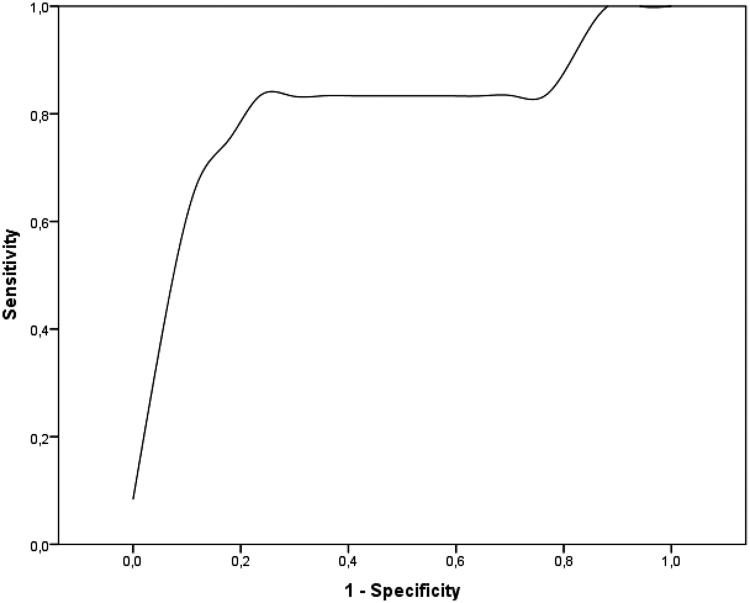
ROC analysis of the changes of RPP between 6 and 12 h after admission.

Below the median of this parameter AKI developed in all cases. If effective RPP was above the median on admission and increased in the first 12 h in the ICU, none of the study participants developed AKI, whereas most patients with decreasing effective RPP did (Figure 1). In most AKI patients, effective RPP decreased between 6 and 12 h. Even though perfusion improved both at 6 and 12 h in the poor RPP group, AKI remained unavoidable (Figure 2). ROC analysis of changes of RPP between 6 and 12 h revealed moderate probability of the RPP for AKI according to both positive and negative likelihood ratios (LR) (1-AUC: 0.804, sensitivity: 86%, specificity: 81%, LR +: 4.50, LR −: 0.18) (Figure 3).

After logistic regression analysis, the change of effective RPP between 6 and 12 h was the only parameter that remained in the equation and that showed an almost significant result (B: −0.091, SE: 0.048, Wald: 3.641, Sig: 0.056, Exp (B): 0.913, 95% CI for Exp (B): 0.813–1.002). The logistic regression analysis with the parameters at other time points or intervals has not revealed significant relationship between basic pressures or RPP and the presence of AKI at 48 h.

## Discussion

The role of arterial blood supply is clear and well-studied, but venous congestion is underestimated in different guidelines, and currently, only the measurement of IAP is emphasized [13,27]. In this paper, having analyzed parameters recorded within 12 h after admission in our study, we are reporting the value of the formula of MAP−(IAP + CVP + Pmean) in predicting AKI beyond ICU admission, incorporating elements of hemodynamic, as well as ventilator-derived and intra-abdominal pressures.

Human studies for investigating the optimal MAP were conducted mostly in patients with septic shock. The generally accepted target is 65 mmHg, a number extrapolated from intraoperative observations and from clinical experience derived from monitoring septic patients’ hemodynamics. Keeping MAP above this value is protective against AKI [28]. From the parameters affecting renal congestion, the elevated IAP (>12 mmHg) is associated with a higher risk of AKI [13]. The CVP (but not the cardiac index) has been shown to be a risk factor of renal failure in cardiac patients and is proved to be independent of IAP [10]. In septic ICU patients, higher CVP and lower diastolic pressure have been associated with AKI [11]. In another study, mean perfusion pressure deficit (defined as MAP − CVP) and diastolic perfusion pressure deficit have been shown to be predictors of worsening kidney function [12]. Against these results, in our prospective study, the median values of MAP were above 70 mmHg, IAP not higher than 12 mmHg and CVP not higher than 8 mmHg at all time-points. To reach these hemodynamic parameters, many patients needed vasopressor therapy and various amounts of intravenous fluids. The aim of our study was to find the simplest, continuously recordable and proper parameter for determining the adequacy of renal perfusion pressure in patients requiring ICU admission after major abdominal surgery.

Mechanical ventilation can increase the risk of AKI, but in our study, we found no differences in the Pmean values between the groups [23]. Because no unique and simple parameter is suitable for monitoring and selecting patients who are at risk of AKI after major abdominal surgery, we tried to find the best combination. In our previous paper, we reported that the MAP−(IAP + CVP + Pmean) formula was the best predictor of AKI on admission to ICU [23]. Moreover, when we combined those parameters even with parameters of ‘normal’ range, we found significant differences between groups in effective RPP at 12 h. We should emphasize that this effect was not due to a change of arterial blood supply as MAP remained unchanged. The factors affecting the venous congestion of the kidney (IAP, CVP, Pmean) have been proved to be independent of each other [23].

The physiological basis of computing these equations was the previously mentioned data concerning venous congestion [10,19]. Medians of all parameters were in so-called ‘normal’ range, and also the CVP was there. There were no significant differences in medians of AKI and of no-AKI groups. The IQRs of MAP are the same across groups at 6 and 12 h, as you can see in Table 2. The IQR of IAP is lower in no-AKI group at 6 h, and this difference disappears at 12 h. The medians and IQRs of CVP are higher in AKI group at both 6 and 12 h, although this difference is not significant statistically. The medians of Pmean are higher in AKI group at both 6 and 12 h (difference is not significant). The net driving pressure of filtration in the glomeruli is about 10 mmHg under physiological condition. The medians and IQRs of the parameters mentioned above are the same range. Therefore, the cumulation of seemingly small changes can result in AKI. CVP represents not only extracapsular, but also the intracapsular pressure of kidney, which can differ because of the tight fibrous capsule of it.

The predictivity of effective RPP was found to be suitable not only on admission but at later time points as well: our prediction model has a sensitivity of 86% and specificity of 81%. When this pressure fell below 40.7 mmHg on admission to the ICU, AKI developed in every case, regardless of any further improvement in perfusion. On the other hand, keeping the effective RPP above its median (40.7 mmHg) on admission and the subsequent 12 h in patients having undergone major abdominal surgery protected against AKI. The individual course of RPP above its median measured on admission to ICU is an important tool in prediction of postoperative AKI and can be a part of the patient's personalized care of the patient. Moreover, monitoring this parameter is simpler and may be much easier than using the points of different scoring systems or models.

Our study has several limitations: first, we had only a relatively small-sized cohort with a low number of subjects recruited from the individual centers. The proportion of patients receiving mechanical ventilation was even smaller and likely received heterogeneous and somewhat different from center-to-center care. Details regarding preexisting or total body fluid excess, vasopressors types and doses or intraoperative blood pressures were not available. In our study, only moderately elevated IAP and a normal range of CVP were found, representing both relative strength and weakness of the study, and our conclusions need to be verified with even sicker patients with higher IAP, Pmean or massive volume overload. The value of effective RPP during renal replacement therapy in the course of AKI remains fully unexplored and likely a fertile territory for future investigations.

## Conclusions

Continuous measurement of MAP, IAP and CVP can be useful in patients with shock and potential intrabdominal hypertension. We were able to demonstrate that computing the effective RPP as [MAP−(IAP + CVP + Pmean)] and combining values even 'near-to-normal’ values of hemodynamic and respiratory parameters can be a predictor of AKI in the early postoperative period. Accordingly, this formula may represent a valuable addition to the care of ICU patients at-risk for AKI. Even if there is a modest elevation of venous pressures then MAP should be kept correspondingly higher to keep RPP above 40.7 mmHg. Additionally, larger studies are needed to explore therapeutic interventions targeting this parameter.

We sincerely appreciated the assistance of Mr. Attila Lénárt-Muszka (Debrecen, Hungary) and of Richard Mann during editing and grammar review.

## Data Availability

The data that support the findings of this study are available from the corresponding author, CsK, upon reasonable request.
